# Structural and functional changes in the microcirculation of lepromatous leprosy patients - Observation using orthogonal polarization spectral imaging and laser Doppler flowmetry iontophoresis

**DOI:** 10.1371/journal.pone.0175743

**Published:** 2017-04-18

**Authors:** Curt Treu, Maria das Graças Coelho de Souza, Omar Lupi, Fernando Lencastre Sicuro, Priscila Alves Maranhão, Luiz Guilherme Kraemer-Aguiar, Eliete Bouskela

**Affiliations:** 1 Laboratório de Pesquisas Clínicas e Experimentais em Biologia Vascular, Centro Biomédico, Universidade do Estado do Rio de Janeiro (UERJ), Rio de Janeiro, Rio de Janeiro, Brazil; 2 Departamento de Dermatologia, Universidade Federal do Estado do Rio de Janeiro (Uni-Rio), Rio de Janeiro, Rio de Janeiro, Brazil; 3 Ambulatório de Obesidade, Policlínica Piquet Carneiro, Departamento de Medicina Interna, Faculdade de Ciências Médicas, Centro Biomédico, Universidade do Estado do Rio de Janeiro, Rio de Janeiro, Brazil; USF Health Morsani College of Medicine, UNITED STATES

## Abstract

Leprosy is a chronic granulomatous infection of skin and peripheral nerves caused by *Mycobacterium leprae* and is considered the main infectious cause of disability worldwide. Despite the several studies regarding leprosy, little is known about its effects on microvascular structure and function *in vivo*. Thus, we have aimed to compare skin capillary structure and functional density, cutaneous vasomotion (spontaneous oscillations of arteriolar diameter), which ensures optimal blood flow distribution to skin capillaries) and cutaneous microvascular blood flow and reactivity between ten men with lepromatous leprosy (without any other comorbidity) and ten age- and gender-matched healthy controls. Orthogonal polarization spectral imaging was used to evaluate skin capillary morphology and functional density and laser Doppler flowmetry to evaluate blood flow, vasomotion and spectral analysis of flowmotion (oscillations of blood flow generated by vasomotion) and microvascular reactivity, in response to iontophoresis of acetylcholine and sodium nitroprusside. The contribution of different frequency components of flowmotion (endothelial, neurogenic, myogenic, respiratory and cardiac) was not statistically different between groups. However, endothelial-dependent and -independent vasodilatations elicited by acetylcholine and sodium nitroprusside iontophoresis, respectively, were significantly reduced in lepromatous leprosy patients compared to controls, characterizing the existence of microvascular dysfunction. These patients also presented a significant increase in the number of capillaries with morphological abnormalities and in the diameters of the dermal papilla and capillary bulk when compared to controls. Our results suggest that lepromatous leprosy causes severe microvascular dysfunction and significant alterations in capillary structure. These structural and functional changes are probably induced by exposure of the microvascular bed to chronic inflammation evoked by the *Mycobacterium leprae*.

## Introduction

Leprosy is a chronic granulomatous infection of skin and peripheral nerves caused by the *Mycobacterium leprae* [1]. This obligatory intracellular pathogen causes nerve damage that affects sensory, motor and autonomic fibers resulting in disabilities and deformities [2,3]. In the last 50 years the prevalence of leprosy has reduced; however, the transmission still occurs and it remains an important public health problem worldwide [4]. According to the World Health Organization, only in 2011, the global incidence of leprosy was 219,075 with India and Brazil leading the number of new cases [5].

Despite the several studies regarding leprosy epidemiology, immunological reactions, peripheral nerve infection and clinical features, little is known about its effects on the microcirculation *in vivo*.

The microcirculation is the part of the vascular bed that encompasses vessels with diameters inferior to100 μm (arterioles, capillaries and venules), where delivery of oxygen and nutrients to tissues and removal of cellular waste as well as the control of peripheral vascular resistance occur [6,7].

Arterioles display spontaneous rhythmic variations of vessel lumen, the so called vasomotion [8] that elicits blood flow oscillations, termed flowmotion, [9] and regulates microvascular blood flow distribution in the skin microvasculature [7].

Investigation of human vasomotion and its consequent flowmotion is possible through the spectral analysis of skin laser Doppler flowmetry (LDF) signal [9]. Moreover, LDF can also be used to investigate endothelial and microvascular function when associated to iontophoresis of acetylcholine (ACh) and sodium nitroprusside (SNP). The iontophoresis allows the transdermal delivery of these vasodilators across the skin using a weak current [10,11]. The subsequent increase in blood flow is, then, recorded by the LDF system.

The analysis of the frequency interval of LDF signal enables the evaluation of five different mechanisms that control flowmotion: endothelial (0.01–0.02 Hz), neurogenic (0.02–0.06 Hz), myogenic, related to vascular smooth muscle cells (VSMC) (0.06–0.15 Hz), respiratory (0.15–0.4 Hz) and cardiac, associated to heart frequency (0.4–1.6 Hz) [12].

The assessment of endothelial and microvascular function in cutaneous microvascular bed constitutes an important noninvasive tool for precocious detection of cardiovascular risk in clinical settings. Since microvascular dysfunction is a systemic process that occurs in a similar manner in the entire body [13,14] it is possible to consider that the impairment of microvascular function observed in the skin microvasculature also occurs in the coronary microcirculation, for example.

The orthogonal polarization spectral (OPS) imaging technique allows real time assessment of the skin microcirculation up to 3 mm in depth [15,16,17,18,19] *in vivo*. This technique enables the evaluation of functional capillary density, capillary morphology, dermal papilla diameter, capillary bulk diameter and capillary diameter.

The present study aimed to assess, *in vivo*, functional and morphological microcirculatory variables in patients with lepromatous leprosy (LL) and healthy age- and gender-matched controls by means of LDF (associated or not to ACh and SNP iontophoresis) and OPS imaging technique in order to evidence microvascular differences between these two groups.

## Materials and methods

This is a cross-sectional study approved by the Ethics Committee of the Hospital Universitário Pedro Ernesto, Universidade do Estado do Rio de Janeiro (2405-CEP/HUPE) performed according to principles outlined in the Declaration of Helsinki (Clinical Trials.gov registration no. NCT02085317).

### Subjects

Ten LL patients under proper treatment were recruited and compared to 10 age- and gender-matched healthy controls. All participants of the study have met the following inclusion criteria and signed the written informed consent.

#### Inclusion criteria

Males with or without lepromatous leprosy, with ages between 20 and 60 years old, body mass index (BMI) between 18 and 29.9 kg/m^2^, Fitzpatrick’s phototype between I and IV [20], able to follow given directions and to attend microvascular assessments and be under treatment for leprosy (for LL patients only) were included in the study.

#### Exclusion criteria

Females, subjects with previously confirmed diagnostic of hypertension, diabetes mellitus, BMI ≥30 kg/m^2^, past or present history of tabagism and ages under 20 and over 60 years old were excluded.

#### Study recruitment

The recruitment occurred between July 20th, 2009 and April 27th, 2012. For LL group, 112 patients were recruited, but only 61 patients came to the laboratory for medical appointment and examinations. From this total, 51 were considered ineligible for the study due to its rigid exclusion criteria. Obesity *per se* was the most common cause for exclusion (18 patients), obesity and hypertension accounted for exclusion of 13 patients, obesity and type II diabetes mellitus were responsible for 9 exclusions and tabagism was the cause of 11 exclusions.

For the control group, 13 volunteers were recruited. However, one was smoker and two were obese and, therefore, ineligible for the study.

### Microvascular assessment

Microvascular assessments occurred from September 8th, 2009 to June 5th, 2012. All participants were asked to arrive at the laboratory after 12 h overnight fast and to abstain from caffeine and alcohol during the last 24 hours. They were accommodated in an acclimatized room (23±1°C) during 20 minutes before microvascular evaluations. All subjects had their anthropometric variables assessed and blood pressure evaluated before the examination to ensure that they met the prerequisites for inclusion.

#### Skin microvascular blood flow and vasomotion

Skin blood perfusion and vasomotion was evaluated by a LDF apparatus (PeriFlux System PF5000, Perimed AB, Stockholm, Sweden) consisting of a transmission of low-power laser light (780 nm) to the tissue through a fiber optic probe that penetrates 0.4–1.0 nm. The light penetration allows the assessment of net red blood cell flow in arbitrary perfusion units (PU) that corresponds to the concentration of moving blood cells and their velocity, in arterioles, capillaries and venules and in anastomosis of deeper blood vessels in dermal layers, within an area of 1 mm^2^ [21]. The LDF signal was recorded continuously during 20 min by an interfaced computer equipped with Perisoft software (PSW 2.50, Perimed AB, Stockholm, Sweden) in order to assess skin blood flow and vasomotion. For these measurements a probe was positioned at the dorsum of the left wrist.

For the fast Fourier transform analysis of LDF signal, the Perisoft software (PSW version 2.50, Perimed AB, Stockholm, Sweden) was used to determine the contribution of different frequency components of flowmotion through the variability of the LDF signal. The frequency spectrum between 0.01 and 1.6 Hz was divided into five frequency intervals: endothelial (0.01–0.02 Hz), neurogenic (0.02–0.06 Hz), myogenic, related to VSMC activity (0.06–0.15 Hz), respiratory (0.15–0.4 Hz) and cardiac, associated to heart frequency (0.4–1.6 Hz) [12,22]. Mean total amplitude value of the total spectrum as well as the mean amplitude values of each frequency interval were recorded and normalized (absolute amplitude at a particular frequency interval divided by the mean amplitude of the entire spectrum) [23]. Normalized results were then compared between controls and LL patients.

#### Iontophoresis of acetylcholine and sodium nitroprusside

Endothelium-dependent and -independent vasodilatations were evaluated by LDF combined to iontophoresis of ACh and SNP, respectively. ACh (Acetylcholine, Sigma-Aldrich, Saint Louis, MO, USA) solution at 1% was delivered by nine iontophoretic pulses of 0.1 mA during 20 s with a 60 s interval to the middle phalanx of the second left finger using an anodal current. On the other hand, SNP (sodium nitroprusside, Niprid^®^ 10mg/ml—Biolab, São Paulo, Brazil) was delivered by seven iontophoretic pulses of 0.2 mA during 20 s with a 180 s interval to the middle phalanx of the third left finger using a cathodal current.

During ACh and SNP iontophoresis, it was possible to evaluate cutaneous blood perfusion (in perfusion units—PUs) at baseline and plateau and vasodilatation expressed in absolute values (difference between plateau and baseline in PUs) and in percentage (% of increase from baseline to plateau). These values were compared between controls and LL patients.

#### Orthogonal Polarization Spectral (OPS) imaging assessment

After acclimatization, cutaneous microcirculation of patients and controls were assessed by OPS imaging (Cytoscan, Cytometrics Inc, Philadelphia, PA, USA) at three different points of the skin lesion area (for LL patients) and of healthy skin area (for controls), according to criteria recommended by De Backer [24]. Images were recorded for 10 seconds at each point and evaluated afterwards using the Cap-Image v7.2 software.

Using OPS imaging, we have evaluated FCD (number of capillaries with flowing red blood cells/mm^2^), DPD (μm, to quantify edema), CD (μm, to detect capillary enlargement), CBD (μm, to assess its degree of change) and CM (percentage of abnormal capillaries per field) of the participants. These variables have already been evaluated in other studies of our group [18,25,26,27].

### Statistical analysis

Clinical and anthropometric variables are presented as mean ± SD and unpaired t test was used to assess statistical differences between them. Results of microcirculatory variables were presented as median [interquartile range] and Mann-Whitney U test was used for comparisons between groups. For all statistical analysis, the Graph Pad Prism 5.0 software (Graph Pad Software Inc., San Diego, CA, USA) was used. *P* value of less than 0.05 was considered significant.

## Results

### Characteristics of control and lepromatous leprosy groups

Table 1 presents anthropometric and clinical characteristics of control and lepromatous leprosy groups. Healthy participants and LL patients did not have obesity or hypertension and there was no significant difference between groups in age, height, weight, BMI and systolic blood pressure (SBP). Although all patients were normotensive, the diastolic blood pressure (DBP) in LL patients was statistically higher compared to controls.

**Table 1:** Clinical and anthropometrical characteristics of groups (mean ±SD).

	Control group (n = 10)	LL group (n = 10)	*P* value
Age (years)	32.7±1.6	33.5±1.8	0.22
Weight (kg)	69.3±9.8	70.1±6.0	0.83
Height (m)	1.73±0.06	1.71±0.05	0.39
BMI (kg/m^2^)	23.1±2.6	24.1±1.8	0.37
SBP (mmHg)	111.2±8.5	117.6±5.6	0.06
DBP (mmHg)	71.2±7.7	78.8±6.0	0.02

LL group—Lepromatous leprosy group; BMI—body mass index, SBP—systolic blood pressure; DBP—diastolic blood pressure.

### Skin microvascular blood flow and flowmotion

At rest, skin blood perfusion was not significantly different between controls and LL patients. Spectral analysis of flowmotion frequency components (endothelial, neurogenic, myogenic, respiratory and cardiac) did not show any significant differences between controls and LL patients. These results are depicted on Table 2.

**Table 2:** Skin microvascular perfusion and flowmotion spectral analysis in controls and in Lepromatous Leprosy (LL) patients measured by laser Doppler flowmetry.

	Controls (n = 10)	LL patients (n = 10)	*P* value
**Total Spectrum (PU)**	5.587 [4.545–7.648]	6.760 [5.051–9.201]	0.393
**Vasomotion (PU/Hz)**			
**Endothelial**	0.4457 [0.3793–0.5214]	0.4077[0.2277–0.4844]	0.481
**Neurogenic**	0.2824 [0.2479–0.3358]	0.2557 [0.2446–0.3295]	0.579
**Myogenic**	0.1477[0.1053–0.2162]	0.1624 [0.1062–0.2955]	0.684
**Respiratory**	0.04968 [0.03397–0.1035	0.06111 [0.03390–0.1314]	0.529
**Cardiac**	0.04462[0.02984–0.06420]	0.06975 [0.05322–0.09371]	0.089

Data are expressed as median [interquartile range]. PU: perfusion units.

### Iontophoresis of acetylcholine and sodium nitroprusside

As demonstrated on Table 3, there are no significant differences in blood flow between controls and LL patients, before ACh and SNP iontophoresis.

**Table 3:** Microvascular measurements before and during iontophoresis using laser Doppler flowmetry.

	Controls (n = 10)	LL patients (n = 10)	*P* value
**ACh-mediated vasodilation**			
**Baseline skin perfusion, PU**	45.52 [32.20–57.73]	46.94 [28.69–58.85]	1.000
**Plateau, PU**	203.4 [165.5–255.4]	70.01 [42.73–78.25]	0.0002
**Number of doses to reach Plateau**	5.50[5.00–7.00]	8.50[8.00–9.00]	0.0003
**Absolute increase, PU**	147.5 [116.6–213.8]	12.40 [8.550–30.15]	0.0001
**Percentage increase, %**	287.4 [247.9–509.8]	46.40 [17.90–83.40]	0.0001
**SNP-mediated vasodilation**			
**Baseline skin perfusion, PU**	29.76 [22.81–56.98]	30.70 [19.59–42.48]	1.000
**Plateau, PU**	145.8 [121.5–194.0]	45.44 [32.35–64.06]	0.0001
**Number of doses to reach Plateau**	5.00[4.00–5.25]	6.50[6.00–7.00]	0.0005
**Absolute increase, PU**	119.1 [85.88–163.7]	13.25 [5.250–21.55]	0.0001
**Percentage increase, %**	376.5 [257.4–564.8]	55.90 [32.98–78.40]	0.0001

Data are expressed as median [interquartile range]. LL: lepromatous leprosy. ACh: acetylcholine. SNP: sodium nitroprusside. PU: perfusion units.

**Fig 1:**
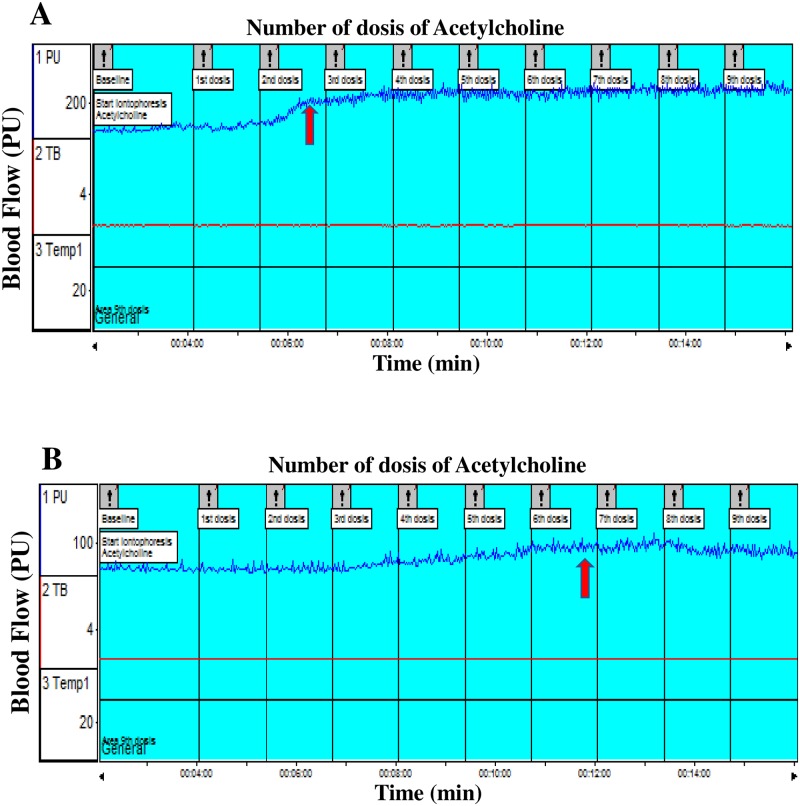
Acetylcholine intophoresis. Microvascular skin blood flow during acetylcholine iontophoresis (A) in controls and (B) in lepromatous leprosy patients. The arrows indicate where the plateau is reached.

During ACh iontophoresis, controls presented a significant increase in microvascular blood flow in relation to LL patients, reaching the plateau with significantly fewer ACh doses (Fig 1). Absolute and percentage values of blood flow, reflecting endothelial-dependent vasodilatation, were significantly higher in controls compared to LL patients (Table 3).

**Fig 2:**
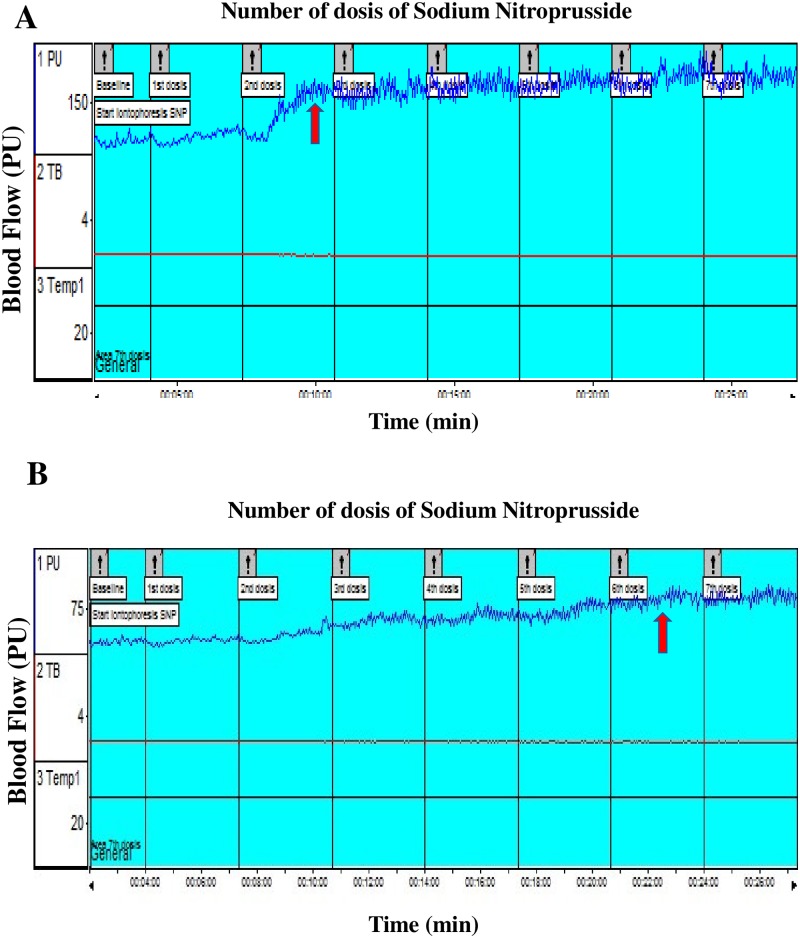
Iontophoresis of sodium nitroprusside. Microvascular skin blood flow during sodium nitroprusside iontophoresis (A) in controls and (B) in lepromatous leprosy patients. The arrows indicate where the plateau is reached.

During SNP iontophoresis, controls presented a significant increase in microvascular blood flow in relation to LL patients, reaching the plateau with significantly fewer SNP doses (Fig 2). Absolute and percentage values of blood flow, corresponding to endothelial-independent vasodilatation, were significantly greater in controls compared to LL patients (Table 3).

### Orthogonal Polarization Spectral (OPS) imaging assessment

Controls and LL patients did not present significant differences concerning FCD and CD. However, LL patients presented a significant increase in DPD, CBD and CM compared to controls (Table 4).

**Table 4:** Orthogonal polarization spectral imaging of skin microcirculation.

	Controls (n = 10)	LL patients (n = 10)	*P* value
**FCD (capillaries/mm**^**2**^**)**	38.38 [36,02–41.95]	39.34 [34.98–46.10]	0.684
**DPD (μm)**	85.73 [81.12–92.59]	100.4 [90.22–112.7]	0.005
**CBD (μm)**	53.01[47.35–56.28]	58.85 [53.99–72.07]	0.043
**CD (μm)**	7.000 [6.538–7.525]	8.033 [6.742–9.542]	0.123
**CM (%)**	6.850 [2.938–10.83]	35.50 [8.750–73.42]	0.014

Data are expressed as median [interquartile range]. LL: lepromatous leprosy. FCD: functional capillary density (number of capillaries with flowing red cells/mm^2^). DPD dermal papilla diameter (μm). CBD, capillary bulk diameter (μm). CD: capillary diameter (μm). CM: capillary morphology (% of abnormal capillaries).

## Discussion

The novel findings of the present study are: (a) LL patients present a significant impairment of endothelium-dependent and -independent microvascular reactivity in response to ACh and SNP iontophoresis, respectively, when compared to controls; (b) LL patients did not present significant changes in none of the five frequency components of flowmotion (endothelial, neurogenic, myogenic, respiratory and cardiac), in comparison to controls and; (c) LL patients presented significant alterations in capillary structure, such as increased DPD, CBD and CM, when compared to controls.

This is the first time, to our knowledge, that an investigation evaluates *in vivo* the impact of LL on structure and function of cutaneous microcirculation. There are, however, two other studies that investigated microvascular blood flow but not microvascular function *per se*. In one study, microvascular blood flow of skin lesions was evaluated by LDF in leprosy patients (not in LL patients) to demonstrate that this noninvasive technique can be useful and clinically acceptable to determine the severity of the hyperemia response, an early indicator of reversal reaction during chemotherapy in these patients [28]. In the other study, the vasomotor reflex, defined by the vasoconstrictor response to autonomic stimuli, was assessed in finger and toe tips by laser Doppler flow temperature technique in order to show skin microvascular dysautonomia in leprosy patients [29].

Turkel and coworkers [30] analyzed, by electron microscopy, the microvascular structure of skin biopsies of LL patients and reported ultrastructural changes in the dermal microvasculature that included: endothelial cell swelling and hypertrophy, increased endothelial cytoplasmic processes, presence of phagocytized, membrane-bound intra-endothelial *M*. *leprae* and perivascular dermal inflammatory infiltrate formed by lymphocytes, macrophages and mast cells. Another study, using immunohistochemical staining, showed that LL patients presented dense and tortuous mesh of microvessels among *M*. *leprae*-glutted macrophages [31]. Based on these observations and considering that endothelial dysfunction precedes structural changes in blood vessels [10] we have speculated that endothelial dysfunction occurs in early stages of LL.

Endothelial cells are key regulators of vascular homeostasis [32]. Due to its localization between blood and VSMC, the endothelium perceives physical and chemical *stimuli* from blood, vascular wall and interstitium and respond with expression and release of several molecules involved in tone regulation, cellular adhesion, coagulation, fibrinolysis, VSMC proliferation and vascular wall inflammation [33].

Endothelial dysfunction is characterized by decreased bioavailability of NO with concomitant increase of endothelium-derived vasoconstrictors, resulting in reduction of endothelium dependent vasodilatation [10,34].

In our study, LL patients presented significant reduction of endothelium- dependent vasodilatation, evidenced by significant impairment of ACh-induced increase in blood flow during iontophoresis in comparison to control group. ACh promotes the release of endothelium-dependent vasodilators including NO [35] produced via endothelial nitric oxide synthase (eNOS or NOS3) activation. Once produced by the endothelium, NO diffuses toward VSMC where it induces cGMP production [36]. Increased levels of cGMP reduce intracellular Ca^2+^ concentration [37] as well as the sensitivity of the contractile apparatus to Ca^2+^ [38], causing relaxation of VSMC.

The significant reduction of ACh-mediated increase in blood flow in LL group, during iontophoresis, can also be explained by the impairment of Lewis triple-flare response (also called nerve axon reflex-mediated vasodilatation). In this mechanism, ACh stimulates the release of the vasodilators calcitonin gene-related peptide and substance P by nociceptive C-type fibers of the somatic peripheral nervous system in the epidermis [14]. Since these fibers are the earliest to be affected by *M*. *leprae* [39], this mechanism of vasodilatation could be compromised in LL patients.

The present study also demonstrated significant impairment of endothelium-independent vasodilatation, during SNP iontophoresis. SNP, an exogenous donor of NO, reacts with sulfhydryl containing compounds in the tissues [40] to produce and release NO, stimulating VSMC relaxation [10].

The observed significant impairment of ACh and SNP-induced increase in blood flow, demonstrated that LL severely inhibited endothelial-dependent and -independent vasodilatory mechanisms, characterizing the presence of microvascular dysfunction in these patients. Since microvascular dysfunction is a systemic phenomenon that occurs in similar way in the entire body [13,14] we can speculate that the impairment of microvascular function observed in the skin microvasculature occurs in other microvascular beds, including the coronary microcirculation.

The analysis of frequency components of flowmotion (endothelial, neurogenic, myogenic, respiratory and cardiac) did not show statistical differences between LL patients and controls.

OPS imaging demonstrated that LL promotes significant alterations in capillary architecture evidenced by increased DPD, CBD and CM compared to controls. Since a significant rise of DPD indicates the onset of the inflammatory process [41], we believe that these structural changes are consequence of immune reactions induced by the disease.

Due to inflammation of peripheral autonomic nerves promoted by *M*. *leprae*, we should expect a significant reduction of the neurogenic component of flowmotion in LL patients, which was not observed even in LL patients with dermal anesthesia. A possible explanation for this could be the diffuse alteration in innervation in LL patients [42]. Thus, we presume that the peripheral autonomic nerves in the skin areas analyzed were not compromised by *M*. *leprae* infection yet.

Another possible reason could be the low reproducibility of the method [43], which demands higher number of participants, in special LL patients, to show possible statistical differences. LL patients, due to social stigma, had difficulties to accept the study invitation and to attend the examinations (we invited 112 patients and only 61 came to the Laboratory). Furthermore, it was difficult to have LL patients eligible for the study due to rigorous inclusion criteria.

On the other hand, despite the small number of LL patients, we have shown an impairment of microvascular reactivity in response to ACh and SNP iontophoresis in the LL group with very expressive statistical significance and significant differences in CBD, DPD and CM in LL patients compared to controls. We considered these data enough to demonstrate the presence of microvascular dysfunction and structural abnormalities in capillaries in LL and decided not to insist in the recruitment of new patients due to the difficulties mentioned above.

In order to exclude any confounding factors from our analysis we did not include hypertensive, diabetic, obese, aged patients and smoker individuals in the study since hypertension [44,45], diabetes mellitus [44,46] obesity [47], age [48,49] and tobacco [50,51,52,53] are factors that alter microvascular reactivity and blood flow evaluated by LDF. OPS imaging has also shown microvascular changes in smokers [54] and hypertensive patients [46].

The interesting findings of this study encouraged our group to investigate whether the impaired microvascular reactivity and capillary morphology abnormalities, observed in LL patients, could also be found in other forms of leprosy, especially in tuberculoid leprosy, the other polar form of the disease.

In conclusion, our results suggest that lepromatous leprosy causes severe microvascular dysfunction and significant alterations in capillary structure. The microcirculatory structural and functional changes found in these patients are probably induced by exposure of the microvascular bed to chronical inflammation evoked by the *Mycobacterium leprae*.

## Supporting information

S1 Table Anthropometric and clinical characteristics of the control group. (DOCX)

S2 Table Anthropometric and clinical characteristics of lepromatous leprosy group. (DOCX)

S3 Table Absolute amplitude of vasomotion frequency components. Controls.
(DOCX)

S4 Table Absolute amplitude of vasomotion frequency components. Lepromatous leprosy patients.
(DOCX)

S5 Table Acetylcholine iontophoresis. Controls.
(DOCX)

S6 Table Acetylcholine iontophoresis. Lepromatous leprosy patients.
(DOCX)

S7 Table Sodium nitroprusside iontophoresis. Controls.
(DOCX)

S8 Table Sodium nitroprusside iontophoresis. Lepromatous leprosy patients.
(DOCX)

S9 Table Orthogonal polarized spectral imaging. Controls.
(DOCX)

S10 Table Orthogonal polarized spectral imaging. Lepromatous leprosy patients.
(DOCX)
