# Supplementary material for: Structural and functional changes in the microcirculation of lepromatous leprosy patients - Observation using orthogonal polarization spectral imaging and laser Doppler flowmetry iontophoresis
Source: PLoS One. 2017 Apr 18;12(4):e0175743. doi: 10.1371/journal.pone.0175743 (PMC5395185; doi:10.1371/journal.pone.0175743)
Supplement: S1 Table — (DOCX) [file pone.0175743.s001.docx]

**S1 Table. Anthropometric and clinical characteristics of the control group.**

| **Participant** | **Age**  **(years)** | **Weight**  **(kg)** | **Height**  **(m)** | **BMI (kg/m2)** | **SBP (mmHg)** | **DBP (mmHg)** |
| --- | --- | --- | --- | --- | --- | --- |
| **1** | 33 | 84.3 | 1.78 | 26.61 | 120 | 82 |
| **2** | 33 | 68.0 | 1.65 | 24.98 | 100 | 60 |
| **3** | 32 | 71.4 | 1.76 | 23.05 | 110 | 70 |
| **4** | 35 | 62.3 | 1.74 | 20.58 | 110 | 70 |
| **5** | 31 | 55.2 | 1.72 | 18.66 | 110 | 70 |
| **6** | 30 | 84.1 | 1.84 | 24.84 | 126 | 80 |
| **7** | 34 | 74.3 | 1.75 | 24.26 | 106 | 60 |
| **8** | 32 | 64.9 | 1.71 | 22.19 | 100 | 70 |
| **9** | 35 | 58.2 | 1.69 | 20.38 | 110 | 70 |
| **10** | 32 | 70.4 | 1.65 | 25.86 | 120 | 80 |
