# Supplementary material for: Structural and functional changes in the microcirculation of lepromatous leprosy patients - Observation using orthogonal polarization spectral imaging and laser Doppler flowmetry iontophoresis
Source: PLoS One. 2017 Apr 18;12(4):e0175743. doi: 10.1371/journal.pone.0175743 (PMC5395185; doi:10.1371/journal.pone.0175743)
Supplement: S2 Table — (DOCX) [file pone.0175743.s002.docx]

**S2 Table. Anthropometric and clinical characteristics of lepromatous leprosy group**

| **Participant** | **Age**  **(years)** | **Weight**  **(kg)** | **Height**  **(m)** | **BMI (kg/m2)** | **SBP (mmHg)** | **DBP (mmHg)** |
| --- | --- | --- | --- | --- | --- | --- |
| **11** | 35 | 61.5 | 1.62 | 23.43 | 110 | 70 |
| **12** | 36 | 71.0 | 1.72 | 24.00 | 120 | 74 |
| **13** | 34 | 75.2 | 1.77 | 24.00 | 120 | 80 |
| **14** | 32 | 73.0 | 1.78 | 23.04 | 118 | 80 |
| **15** | 32 | 62.0 | 1.7 | 21.45 | 110 | 80 |
| **16** | 31 | 75.2 | 1.7 | 26.02 | 120 | 70 |
| **17** | 36 | 62.0 | 1.7 | 21.45 | 110 | 80 |
| **18** | 32 | 76.0 | 1.75 | 24.82 | 122 | 86 |
| **19** | 33 | 74.8 | 1.7 | 25.88 | 126 | 88 |
| **20** | 34 | 70.4 | 1.63 | 26.50 | 120 | 80 |
