# Supplementary material for: Structural and functional changes in the microcirculation of lepromatous leprosy patients - Observation using orthogonal polarization spectral imaging and laser Doppler flowmetry iontophoresis
Source: PLoS One. 2017 Apr 18;12(4):e0175743. doi: 10.1371/journal.pone.0175743 (PMC5395185; doi:10.1371/journal.pone.0175743)
Supplement: S3 Table — Controls. (DOCX) [file pone.0175743.s003.docx]

**S3 Table. Absolute amplitude of vasomotion frequency components. Controls.**

| **Participant** | **Endothelial** | **Neurogenic** | **Myogenic** | **Respiratory** | **Cardiac** |
| --- | --- | --- | --- | --- | --- |
| **1** | 2.38400000 | 1.48000 | 0.84410000 | 0.300860 | 0.221612000 |
| **2** | 2.80033333 | 1.83060 | 0.54403333 | 0.222260 | 0.169508620 |
| **3** | 1.80600000 | 1.57120 | 0.58603333 | 0.182820 | 0.223801720 |
| **4** | 3.43000000 | 1.44540 | 0.62186667 | 0.124520 | 0.148577590 |
| **5** | 1.16366667 | 0.74500 | 0.44040000 | 0.170160 | 0.123905170 |
| **6** | 2.48933330 | 1.79000 | 1.75983333 | 0.851240 | 0.563370690 |
| **7** | 3.24566667 | 1.88740 | 1.77730000 | 0.822220 | 0.497137930 |
| **8** | 4.66700000 | 3.68780 | 1.28323333 | 0.327780 | 0.377370690 |
| **9** | 1.25633333 | 1.42120 | 0.99840000 | 0.547740 | 0.379896550 |
| **10** | 3.23333330 | 1.48000 | 0.54190000 | 0.194760 | 0.157162070 |
