# Supplementary material for: Structural and functional changes in the microcirculation of lepromatous leprosy patients - Observation using orthogonal polarization spectral imaging and laser Doppler flowmetry iontophoresis
Source: PLoS One. 2017 Apr 18;12(4):e0175743. doi: 10.1371/journal.pone.0175743 (PMC5395185; doi:10.1371/journal.pone.0175743)
Supplement: S4 Table — Lepromatous leprosy patients. (DOCX) [file pone.0175743.s004.docx]

**S4 Table. Absolute amplitude of vasomotion frequency componentes. Lepromatous leprosy patients.**

| **Participant** | **Endothelial** | **Neurogenic** | **Myogenic** | **Respiratory** | **Cardiac** |
| --- | --- | --- | --- | --- | --- |
| **11** | 1.80433333 | 1.6426 | 2.6301 | 1.30852 | 0.95880172 |
| **12** | 1.53066667 | 2.3698 | 3.1106 | 1.6786 | 0.89467241 |
| **13** | 2.79066667 | 1.46 | 1.01196667 | 0.51984 | 0.3894569 |
| **14** | 2.229 | 2.5222 | 2.78183333 | 1.1833 | 0.91293966 |
| **15** | 1.997 | 0.907 | 0.33613333 | 0.1854 | 0.20889655 |
| **16** | 3.43233333 | 1.8242 | 1.18196667 | 0.3996 | 0.51087069 |
| **17** | 3.28566667 | 3.4942 | 1.3382 | 0.27482 | 0.67993966 |
| **18** | 2.79433333 | 2.1134 | 0.65243333 | 0.17992 | 0.1495 |
| **19** | 0.79433333 | 0.637 | 0.45423333 | 0.14836 | 0.15305172 |
| **20** | 2.84 | 1.7656 | 0.50083333 | 0.19338 | 0.22332759 |
