# Supplementary material for: Structural and functional changes in the microcirculation of lepromatous leprosy patients - Observation using orthogonal polarization spectral imaging and laser Doppler flowmetry iontophoresis
Source: PLoS One. 2017 Apr 18;12(4):e0175743. doi: 10.1371/journal.pone.0175743 (PMC5395185; doi:10.1371/journal.pone.0175743)
Supplement: S5 Table — Controls. (DOCX) [file pone.0175743.s005.docx]

**S5 Table. Acetylcholine Iontophoresis. Controls.**

| **Participant** | **Baseline (mean PU)** | **Plateau (doses)** | **Plateau (mean PU)** | **Increase Baseline-Plateau (PU)** | **% Increase Baseline-Plateau** |
| --- | --- | --- | --- | --- | --- |
| **1** | 55.66 | 5 | 208.47 | 152.8 | 274.5 |
| **2** | 20.45 | 6 | 72.49 | 52.0 | 254.5 |
| **3** | 59.24 | 5 | 201.33 | 142.1 | 239.9 |
| **4** | 35.74 | 4 | 205.39 | 169.7 | 474.7 |
| **5** | 32.96 | 7 | 174.79 | 141.8 | 430.3 |
| **6** | 70.96 | 5 | 284.05 | 213.1 | 300.3 |
| **7** | 29.92 | 7 | 245.82 | 215.9 | 721.6 |
| **8** | 51.32 | 6 | 179.93 | 128.6 | 250.6 |
| **9** | 39.72 | 5 | 284.02 | 244.3 | 615.1 |
| **10** | 57.22 | 7 | 137.80 | 80.6 | 140.8 |
