# Supplementary material for: Structural and functional changes in the microcirculation of lepromatous leprosy patients - Observation using orthogonal polarization spectral imaging and laser Doppler flowmetry iontophoresis
Source: PLoS One. 2017 Apr 18;12(4):e0175743. doi: 10.1371/journal.pone.0175743 (PMC5395185; doi:10.1371/journal.pone.0175743)
Supplement: S6 Table — Lepromatous leprosy patients. (DOCX) [file pone.0175743.s006.docx]

**S6 Table. Acetylcholine Iontophoresis. Lepromatous leprosy patients.**

| **Participant** | **Baseline (mean PU)** | **Plateau (doses)** | **Plateau (mean PU)** | **Increase Baseline-Plateau (PU)** | **% Increase Baseline-Plateau** |
| --- | --- | --- | --- | --- | --- |
| **11** | 62.06 | 8 | 73.3 | 11.2 | 18.1 |
| **12** | 57.78 | 8 | 66.72 | 8.9 | 15.5 |
| **13** | 13.79 | 7 | 18.86 | 5.1 | 36.8 |
| **14** | 51.42 | 9 | 80.24 | 28.8 | 56.0 |
| **15** | 41.53 | 9 | 49.01 | 7.5 | 18.0 |
| **16** | 64.26 | 9 | 75.59 | 11.3 | 17.6 |
| **17** | 33.65 | 9 | 56.8 | 23.2 | 68.8 |
| **18** | 10.33 | 8 | 23.87 | 13.5 | 131.1 |
| **19** | 50.45 | 9 | 99.62 | 49.2 | 97.5 |
| **20** | 43.42 | 8 | 77.58 | 34.2 | 78.7 |
