# Supplementary material for: Structural and functional changes in the microcirculation of lepromatous leprosy patients - Observation using orthogonal polarization spectral imaging and laser Doppler flowmetry iontophoresis
Source: PLoS One. 2017 Apr 18;12(4):e0175743. doi: 10.1371/journal.pone.0175743 (PMC5395185; doi:10.1371/journal.pone.0175743)
Supplement: S7 Table — Controls. (DOCX) [file pone.0175743.s007.docx]

**S7 Table. Sodium Nitroprusside Iontophoresis. Controls.**

| **Participant** | **Baseline (mean PU)** | **Plateau (doses)** | **Plateau (mean PU)** | **Increase Baseline-Plateau (PU)** | **% Increase Baseline-Plateau** |
| --- | --- | --- | --- | --- | --- |
| **1** | 27.28 | 5 | 104.67 | 77.4 | 283.7 |
| **2** | 25.29 | 5 | 141.84 | 116.6 | 460.9 |
| **3** | 77.8 | 4 | 287.97 | 210.2 | 270.1 |
| **4** | 30.8 | 4 | 180.08 | 149.3 | 484.7 |
| **5** | 15.38 | 6 | 94.26 | 78.9 | 512.9 |
| **6** | 32.42 | 4 | 127.08 | 94.7 | 292.0 |
| **7** | 28.72 | 5 | 235.65 | 206.9 | 720.5 |
| **8** | 55.51 | 5 | 177.14 | 121.6 | 219.1 |
| **9** | 14.76 | 5 | 142.04 | 127.3 | 862.3 |
| **10** | 61.37 | 6 | 149.52 | 88.2 | 143.6 |
