# Supplementary material for: Structural and functional changes in the microcirculation of lepromatous leprosy patients - Observation using orthogonal polarization spectral imaging and laser Doppler flowmetry iontophoresis
Source: PLoS One. 2017 Apr 18;12(4):e0175743. doi: 10.1371/journal.pone.0175743 (PMC5395185; doi:10.1371/journal.pone.0175743)
Supplement: S8 Table — Lepromatous leprosy patients. (DOCX) [file pone.0175743.s008.docx]

**S8 Table. Sodium Nitroprusside Iontophoresis. Lepromatous leprosy patients.**

| **Participant** | **Baseline (mean PU)** | **Plateau (doses)** | **Plateau (mean PU)** | **Increase Baseline-Plateau (PU)** | **% Increase Baseline-Plateau** |
| --- | --- | --- | --- | --- | --- |
| **11** | 35.65 | 6 | 35.58 | -0.1 | -0.2 |
| **12** | 77.8 | 6 | 80.52 | 2.7 | 3.5 |
| **13** | 8.2 | 6 | 14.34 | 6.1 | 74.9 |
| **14** | 40.87 | 7 | 60.3 | 19.4 | 47.5 |
| **15** | 28.75 | 7 | 41.05 | 12.3 | 42.8 |
| **16** | 27.96 | 7 | 56.56 | 28.6 | 102.3 |
| **17** | 20.81 | 7 | 33.11 | 12.3 | 59.1 |
| **18** | 32.64 | 7 | 49.83 | 17.2 | 52.7 |
| **19** | 47.32 | 6 | 75.33 | 28.0 | 59.2 |
| **20** | 15.92 | 6 | 30.08 | 14.2 | 88.9 |
