# Supplementary material for: Structural and functional changes in the microcirculation of lepromatous leprosy patients - Observation using orthogonal polarization spectral imaging and laser Doppler flowmetry iontophoresis
Source: PLoS One. 2017 Apr 18;12(4):e0175743. doi: 10.1371/journal.pone.0175743 (PMC5395185; doi:10.1371/journal.pone.0175743)
Supplement: S9 Table — Controls. (DOCX) [file pone.0175743.s009.docx]

**S9 Table. Orthogonal polarized spectral imaging. Controls.**

| **Participant** | **FCD 1** | **FCD 2** | **FCD 3** | **DPD 1** | **DPD 2** | **DPD 3** | **CBD 1** | **CBD 2** | **CBD 3** | **DC 1** | **DC 2** | **DC 3** | **CM 1** | **CM 2** | **CM 3** |
| --- | --- | --- | --- | --- | --- | --- | --- | --- | --- | --- | --- | --- | --- | --- | --- |
| **1** | 31.29 | 39.83 | 28.45 | 81.9 | 82.6 | 88.8 | 59.5 | 36.7 | 66.2 | 5.6 | 6.8 | 8.3 | 8.0 | 0.0 | 1.0 |
| **2** | 45.5 | 34 | 45.5 | 98.5 | 78.4 | 67.0 | 83.5 | 33.5 | 30.4 | 9.2 | 6.3 | 6.4 | 6.0 | 25 | 6.0 |
| **3** | 19.91 | 22.76 | 39.83 | 90.1 | 101.6 | 84.3 | 75.1 | 65.6 | 59.1 | 6.8 | 8.9 | 8.9 | 0.0 | 0.0 | 0.0 |
| **4** | 36.98 | 45.51 | 42.67 | 66.6 | 82.8 | 92.3 | 46.3 | 47.9 | 62.0 | 6.4 | 5.4 | 8.3 | 18.0 | 0.0 | 13.0 |
| **5** | ------- | 34.05 | 51.2 | ------ | 75.1 | 91.2 | ------ | 42.2 | 65.7 | ------ | 5.7 | 6.8 | ------ | 0.0 | 5.5 |
| **6** | 31.29 | 45.51 | 51.2 | 98.7 | 107.1 | 77.3 | 61.7 | 62.5 | 41.3 | 5.6 | 7.9 | 7.9 | 11.0 | 6.3 | 0.0 |
| **7** | 54.05 | 31.29 | 31.29 | 95.7 | 89.9 | 85.6 | 49.2 | 41.5 | 40.1 | 8.1 | 5.7 | 11.4 | 0.0 | 0.0 | 11.0 |
| **8** | 42.6 | 34.14 | 36.9 | 85 | 71.5 | 69.6 | 30.6 | 45.0 | 27.0 | ------ | 7.9 | 6.3 | 0.0 | 31.6 | 30.7 |
| **9** | 31.3 | 31.3 | 48.3 | 73.2 | 88.9 | 99.0 | 54.7 | 34.9 | 89.3 | 3.4 | 8.3 | 5.1 | 0.0 | 18 | 5.8 |
| **10** | 39.8 | 36.9 | 36.9 | 105 | 117.3 | 69.9 | 51.2 | 60.4 | 34.2 | 5.4 | 8.4 | 6.1 | 13.0 | 15 | 0.0 |

**Legend**

| **FCD** |  | Functional Capillary Density |  |
| --- | --- | --- | --- |
| **DPD** |  | Dermal Papilla Diameter |  |
| **CBD** |  | Capillary Bulk Diameter |  |
| **DC** |  | Capillary Diameter |  |
| **CM** |  | Capilllary Morphology |  |
