# Supplementary material for: Structural and functional changes in the microcirculation of lepromatous leprosy patients - Observation using orthogonal polarization spectral imaging and laser Doppler flowmetry iontophoresis
Source: PLoS One. 2017 Apr 18;12(4):e0175743. doi: 10.1371/journal.pone.0175743 (PMC5395185; doi:10.1371/journal.pone.0175743)
Supplement: S10 Table — Lepromatous leprosy patients. (DOCX) [file pone.0175743.s010.docx]

**S10 Table. Orthogonal polarized spectral imaging. Lepromatous leprosy patients.**

| **Participant** | **FCD 1** | **FCD 2** | **FCD 3** | **DPD 1** | **DPD 2** | **DPD 3** | **CBD 1** | **CBD 2** | **CBD 3** | **DC 1** | **DC 2** | **DC 3** | **CM 1** | **CM 2** | **CM 3** |
| --- | --- | --- | --- | --- | --- | --- | --- | --- | --- | --- | --- | --- | --- | --- | --- |
| **11** | 38.45 | 39.91 | 32.76 | ------ | ------ | 111.8 | ------ | 91.5 | 51.3 | ------ | 19.0 | 8.5 | ------- | 42.0 | 25.0 |
| **12** | 32.76 | 34.14 | 39.91 | 95 | 85.6 | 119.0 | 52.8 | 44.4 | 79.0 | 8.5 | 7.9 | 11.5 | 50.0 | 33.0 | 57.0 |
| **13** | 42.67 | 31.29 | 32.76 | 75.8 | 116.3 | 110.4 | 38.4 | 76.4 | 62.1 | 7.4 | 5.6 | 6.1 | 0.0 | 9.0 | 0.0 |
| **14** | 36.9 | 54 | 34 | 93.7 | 174.0 | 144.5 | 43.6 | 86.0 | 92.6 | 4.7 | 6.0 | 6.8 | 100.0 | 58 | 58.0 |
| **15** | 34.2 | 31.3 | 34.14 | 95.4 | 85.8 | 84.6 | 60.8 | 59.2 | 56.2 | 6.1 | 8.2 | 10.1 | 20.0 | 54 | 38.5 |
| **16** | 75.4 | 25.6 | 42.7 | 132.6 | 135.7 | 77.8 | 103.1 | 96.3 | 32.3 | 14.5 | 7.4 | 6.7 | 100.0 | 100.0 | 46.7 |
| **17** | 28.4 | 31.3 | 34.1 | 104.5 | 125.2 | 86.4 | 45.8 | 71.2 | 49.7 | 6.8 | 7.5 | 9.5 | 0.0 | 9 | 0.0 |
| **18** | 31.3 | 54 | 45.5 | 101.2 | 79.3 | 91.8 | 46.6 | 47.2 | 54.0 | 5.6 | 4.7 | 10.3 | 18.0 | 16.7 | 56.0 |
| **19** | 45.5 | 51.2 | 39.8 | 117.9 | 78.4 | 95.3 | 95.2 | 39.3 | 70.6 | 11.2 | 9.6 | 7.9 | 50.0 | 83.0 | 100.0 |
| **20** | 48.4 | 65.4 | 36.9 | 92.9 | 77.2 | 81.5 | 38.6 | 45.0 | 44.2 | ------- | 7.0 | 8.4 | 0.0 | 17 | 15.0 |

**Legend**

| **FCD** |  | Functional Capillary Density |  |
| --- | --- | --- | --- |
| **DPD** |  | Dermal Papilla Diameter |  |
| **CBD** |  | Capillary Bulk Diameter |  |
| **DC** |  | Capillary Diameter |  |
| **CM** |  | Capilllary Morphology |  |
